# Epigenetic Basis of Lead-Induced Neurological Disorders

**DOI:** 10.3390/ijerph17134878

**Published:** 2020-07-07

**Authors:** Tian Wang, Jie Zhang, Yi Xu

**Affiliations:** School of Food and Bioengineering, Hefei University of Technology, Hefei 230009, China; haloTianWang@163.com (T.W.); 17354278037@163.com (J.Z.)

**Keywords:** epigenetics, lead exposure, neurological disorders, DNA methylation, histone methylation, histone acetylation, circRNA

## Abstract

Environmental lead (Pb) exposure is closely associated with pathogenesis of a range of neurological disorders, including Alzheimer’s disease (AD), Parkinson’s disease (PD), amyotrophic lateral sclerosis (ALS), attention deficit/hyperactivity disorder (ADHD), etc. Epigenetic machinery modulates neural development and activities, while faulty epigenetic regulation contributes to the diverse forms of CNS (central nervous system) abnormalities and diseases. As a potent epigenetic modifier, lead is thought to cause neurological disorders through modulating epigenetic mechanisms. Specifically, increasing evidence linked aberrant DNA methylations, histone modifications as well as ncRNAs (non-coding RNAs) with AD cases, among which circRNA (circular RNA) stands out as a new and promising field for association studies. In 23-year-old primates with developmental lead treatment, Zawia group discovered a variety of epigenetic changes relating to AD pathogenesis. This is a direct evidence implicating epigenetic basis in lead-induced AD animals with an entire lifespan. Additionally, some epigenetic molecules associated with AD etiology were also known to respond to chronic lead exposure in comparable disease models, indicating potentially interlaced mechanisms with respect to the studied neurotoxic and pathological events. Of note, epigenetic molecules acted via globally or selectively influencing the expression of disease-related genes. Compared to AD, the association of lead exposure with other neurological disorders were primarily supported by epidemiological survey, with fewer reports connecting epigenetic regulators with lead-induced pathogenesis. Some pharmaceuticals, such as HDAC (histone deacetylase) inhibitors and DNA methylation inhibitors, were developed to deal with CNS disease by targeting epigenetic components. Still, understandings are insufficient regarding the cause–consequence relations of epigenetic factors and neurological illness. Therefore, clear evidence should be provided in future investigations to address detailed roles of novel epigenetic factors in lead-induced neurological disorders, and efforts of developing specific epigenetic therapeutics should be appraised.

## 1. Introduction

Lead/Pb is a xenobiotic metal continuing to threaten human health in a global perspective [1]. Lead is a ubiquitous toxic pollutant, and can enter the human body through a variety of exposure routes. Recent years have witnessed the removal of lead from paints and gasoline; however, it can still be found in a range of daily products, including toys, batteries, food and water. Among them, the major risks were accounted for by inhalation of air contaminated with lead dust, as well as ingestion of contaminated food or water [2,3,4]. Lead exposure can be detrimental to every human organ, but its toxicity profoundly pervades in brains, especially in early developmental years. In human studies, lead neurotoxicity displayed a robust gender differences, which has been documented in studies involving spatial memory, motor behavior, brain gene expression and dopamine metabolism [5,6]. Of note, although gender differences in lead neurotoxicity have long been reported, very little attention was paid to explore the underlying mechanisms [3].

Lead can cause a variety of adverse outcome on central nervous system (CNS), and is regarded as an important environmental insult leading to multiple neurological disorders, such as Alzheimer’s disease (AD), Parkinson’s disease (PD), amyotrophic lateral sclerosis (ALS), attention deficit/hyperactivity disorder (ADHD), etc. [7,8,9,10,11,12]. Lead is a risk factor of CNS-related diseases even with a very low exposure level, and the current “safe” level is 5 mg/dl for children, as proposed by Centers for Disease Control and Prevention (CDC) [13]. Despite this, cognitive impairment below that dosage has been suggested, implying that “no level of lead exposure is safe” [14,15].

Recent researches suggest the importance of epigenetic mechanisms in defining the relations between lead exposure and etiology of neurological diseases [16]. Epigenetics are heritable changes in gene expression that are not relevant to alterations in the genetic code [3]. The broadly studied epigenetics can be classified into three forms: DNA methylation, histone tail modification (or histone modification), and non-coding RNA (ncRNA) [17,18,19]. These forms were associated with pathogenesis of various CNS diseases [20]. Meanwhile, lead is recognized as an epigenetic disruptor, based on its capacity to drive epigenetic changes in the context of neural development and synaptic plasticity [14]. This fast-moving field of epigenetics opened an avenue for understanding how environmentally toxic signals like lead exposure could be readily sensed by organisms and relayed to reprogram gene expression profiles, consequently resulting in neural impairment as well as diseases. In particular, epigenetics are optimal candidates to explain “fetal origin, late onset” characteristics of Alzheimer’s disease [3]. Although only a limited amount of evidence directly suggested an epigenetic basis in lead-induced neurological disorders, epigenetic factors may still constitute important molecular aspects connecting environmental lead exposure and CNS-related disease.

In this review, we summarized epigenetic advances involved in pathogenesis of AD, PD, ALS and ADHD, as well as their relevance with lead exposure. Among epigenetic elements, DNA CpG (cytosine-phosphate-guanine) methylation receives the most extensive attention, with novel histone modifications and ncRNA forms are proposed as a newly evolving perspective. In addition, we discussed therapeutic approaches targeting epigenetic molecules to reprogram disease pathogenesis.

## 2. Alzheimer’s Disease

### 2.1. AD and Risk Genes

Alzheimer’s disease is a highly prevalent and progressive neurodegenerative disease [16]. AD is pathologically characterized by senile plaques neurofibrillary tangles, gradual memory loss and difficulty in performing normal activities. AD is a complex syndrome, with etiology from both genetic and environmental factors, while the sporadic nature of most AD cases supports an environmental relevance that manifests progressive characteristics [21,22]. Moreover, late onset Alzheimer’s disease (LOAD) constitutes the majority of AD cases (~90%) and has no clear genetic connection. LOAD can be induced by infantile lead exposure, resulting in a “fetal origin, late onset” phenomenon [3].

Some genes were identified as risk factors accounting for AD pathogenesis, such as amyloid precursor protein (*APP*), presenilin 1 (*PSEN1*), presenilin 2 (*PSEN2*), apolipoprotein E (*APOE*) [16]. About 1% of AD is caused by mutations of *APP*, *PSEN1* or *PSEN2*, which are involved in the production of the Aβ peptide [23]. *APOE* gene represents the major genetic risk factor for LOAD and then its mutation is linked to most AD cases, accounting for seven novel variants in 29 mild cognitive impairment subjects [24]. Furthermore, β-site amyloid precursor protein cleaving enzyme 1 (*BACE1*) encodes a β-secretase involved in AβPP (β-site amyloid precursor protein) cleavage, and specific protein 1 (*SP1*) encodes a transcription factor responsible for regulation of *AβPP* and *BACE1* expression. The majority of these genes participated in Aβ formation and dynamics [22]. The risk gene may be susceptible to lead invasion as well as epigenetic regulation, thereby involved in epigenetic connection with AD development.

### 2.2. AD and Lead Exposure

Numerous metal exposures can result in the occurrence of AD, ranging from aluminum, zinc, mercury to lead [8,25,26]. A growing body of evidence supported close relations of AD patients and higher lead exposure. For population-based evidence, it was found that higher cumulative lead levels were associated with decreased memory levels [27]. In 2012, Lee et al. examined the blood lead level (BLL) of 80 AD patients and 130 healthy people in a hospital of Seoul. According to the results, average BLLs were 0.20 µg/dl and 0.17 µg/dl for AD patients and healthy participants, respectively [28]. Fathabadi et al. carried out another case-control study from the patients referring to Imam Reza Hospitals of Birjand, Iran in 2016 to 2017. For comparison, the average BLL in AD case group was 22.22 mg/dL, which was significantly higher than controls [29]. In a recent epidemiological review, early life lead exposure was recognized as a potential risk factor for AD in the Mexican population [26]. Despite this, case-control studies are not well suited to accommodate long latency between exposure and disease onset, but still, a new cohort study focusing on the longitudinal association between BLL and AD mortality was performed in 2019. After examining 8080 elders (over 60 years old) with BLL data from US National Health and Nutrition Examination Survey (1999 to 2008), the authors concluded that BLL had a positive, albeit not statistically significant, association with AD mortality [30].

In addition to population-based research, multiple animal models were established to investigate environmental etiology of AD. In primates exposed to lead during first two-months of life, significant adverse brain alterations were observed at 23 years of age, along with increased amyloidogenesis and senile plaque deposition, during which key genes in the amyloid processing pathway were upregulated [31]. Similarly, rats with early lead treatment exhibited increased expression of *APP* mRNA and elevated Aβ aggregation when rats were grown to 20 months of age [32]. These animal trials demonstrated that lead is a risk factor of AD.

### 2.3. Epigenetic Basis of AD

Epigenetic evidence in AD pathogenesis is found in human studies of various tissues, animal trials and in vitro models [16]. Epigenetic changes associated with AD were observed in DNA methylation, histone modifications and ncRNA. In a human postmortem case-control study, global DNA methylation was reduced in the entorhinal cortex of AD subjects by quantifying 5-methylcytosine (5-mC) [33]. For a single twin pair discordant for AD, DNA from the temporal neocortex was hypomethylated from AD patients comparing to their healthy twins, suggesting that global hypomethylation may co-exist in CNS with AD symptoms in an aging population [34]. Genome-wide methylation studies in AD brain tissues revealed that hundreds of genetic regions were differentially methylated compared with healthy brains. Apart from CNS, AD peripheral tissues also showed methylation dysregulation, providing potential circulating markers for AD diagnosis [35,36,37,38]. It is noteworthy that age and gender should be taken into consideration in explaining and integrating methylation data deriving from different studies.

DNA CpG methylation predominantly exerts its function via moderating the expression of target genes, thus the methylation level at specific genetic loci may be critical in determining its physiological outcome. In terms of AD, the *APP* (amyloid precursor protein) gene was found to be rich in CpG nucleotides, along with other AD-related genes predisposed to be reprogrammed by causative agents [22]. Fuso et al.’s study was consistent with these findings, discovering that DNA methylation status was associated with consequent deregulation of *PSEN1* and *BACE1*, which encode proteins contributing to Aβ production and accumulation in cultured cell model [39]. Interestingly, there are conflicting results describing the methylation status of AD-related genes. By examining specific CpG sites in blood DNA or postmortem brain regions, none of the major AD genes, such as *APP*, *PSEN1* and *PSEN2*, were consistently differentially methylated in AD samples relative to controls [40,41,42,43,44]. These discrepancies may be attributed to limited number of postmortem samples, age difference and inconsistency between circulating markers and CNS molecules. In 2015, Hannon et al. assessed epigenome-wide association studies (EWAS) using whole blood tissues and brain tissues from 122 individuals. They concluded that, for the majority of methylation sites, interindividual variation in whole blood is not a strong predictor of cases in the brain, but still, the utility of blood-based EWAS to identify epigenetic markers of disease should not be discounted [45]. In addition to risk genes, *TREM2* (Triggering receptor expressed on myeloid cells 2) and *BDNF* (brain-derived neurotrophic factor) are among the most replicated genes differentially methylated in AD specimens, providing the potentially valid epigenetic biomarkers for AD [46,47,48].

5-hydroxymethylcytosine (5-hmC) is a newly described epigenetic modification and regarded as product of 5-mC (5-methylcytosine) oxidation [49]. Different from 5-mC, 5-hmC is selectively present in promoter regions of genes with lower CpG content and mostly correlates with gene expression instead of repression. Recent studies indicated an increase of 5-hmC level in aging mouse brain, implicating 5-hmC enrichment in neurodegeneration [50,51,52]. Ellison et al. attempted to measure 5-hmC profiles using postmortem brains classified into varying stages of disease progression. The authors revealed that 5-hmC levels were elevated in preclinical AD subjects across the tested brain regions; however, these alterations returned to normal when specimen from progressive stage of AD was examined [53].

While CpG methylation is stable and can even persist into the next generation, histone tail modifications were relatively dynamic and more prone to environmental signals [54]. Based on studies in AD patients, major histone-modifying forms like acetylation, methylation and phosphorylation were implicated in AD pathogenesis, as determined in AD brain and peripheral blood [55]. In terms of histone acetylation, the investigation of transgenic mouse models of AD revealed an early increase of H4K12ac (acetylation of histone H4 at lysine 12). Nonetheless, this increase was not observed in AD patients, showing a dynamic association with early stage of manifestations [56]. In the temporal lobe of AD subjects, mass spectrometry revealed a significantly decreased H3K18/K23ac (acetylation of histone H3 at lysine 18/23) level in AD samples compared to controls [57]. In another instance, hypoacetylation of H4, but not H3, was observed in tg2576 mice, a model of amyloid pathology [58]. Therefore, altered acetylation showed some extent of site specificity in a range of AD pathology-like models. Interestingly, while HDACs (histone deacetylases) contribute to deacetylation process of histone marks regardless of acetylated residues, HDAC2 (histone deacetylase 2) levels were increased in AD samples [59], and the administration of various pan-HDAC inhibitors (HDACis) was able to restore associative memory in APP/PS1D9 mice, a model for AD-like pathology [60,61]. Although mounting evidence suggested divergent regulation towards a distinct histone acetylation site, a recent systematic analysis showed that there are no consensus findings regarding the roles of global H3 or H4 acetylation in AD [62].

In contrast with substantial evidence of histone acetylation, correlations of AD with histone methylation were less suggested. Walker et al. cultured hippocampal/cortical neurons from 3xTg-AD mouse model and quantified H3K9 methylation levels across the lifespan [63]. According to the findings, H3K9me (methylation of histone H3 at lysine 9) increased with age in non-transgenic neurons, a phenomenon further amplified in AD model neurons. Moreover, a higher H3K9me3 (trimethylation of histone H3 at lysine 9) level was connected to the downregulation of *BDNF* gene expression [63]. Histone methylation shows clear site specificity in modulatory effect, for instance, H3K4me3 (trimethylation of histone H3 at lysine 4) and H3K27me3 (trimethylation of histone H3 at lysine 27) activates or inhibits gene expression, respectively. First discovered in embryonic stem cells, H3K4me3 and H3K27me3 co-occurred in unique genetic location, forming an epigenetic state called “bivalency” [64]. A recent genome-wide methylation study in AD brains showed that the identified DMRs (differentially methylated regions) overlapped promoters enriched by bivalent histone marks, indicating that the bivalent region was susceptible for remodeling in AD pathogenesis [37].

Apart from these routine modifications, an elevated phosphorylation of H3 was revealed in the frontal cortex of AD patient brains [65]. For site-specific modification, hyperphosphorylation of H3 Ser10 was described in AD hippocampal neurons, and this mark can only be found in the postmitotic neuronal cytoplasm (not nucleus) [66]. Besides, Tao et al. reported a finding concerning SUMOylation (SUMO, small ubiquitin-like modifiers) in neurodegeneration, demonstrating that, in a mouse AD model, SUMOylation of HDAC1 was a protective mechanism against Aβ toxicity [67].

ncRNAs are epigenetic regulators extensively investigated in brains and peripheral tissues of patients suffering from neurological disorders, as well as in animal and in vitro models [68,69,70]. Among diverse forms of ncRNAs, roles of microRNAs (miRNAs) were greatly appreciated. The target miRNAs were primarily selected for association studies due to their sequence complementarity with AD-related genes. Regarding Aβ genesis, increased *BACE1* expression was reported in sporadic AD brains [71]. The miR-29 family was linked to *BACE1* regulation in vivo, showing marked reductions in patients suffering from AD or other forms of dementia [72]. The reductions were accompanied with exceptionally high BACE1 protein levels, and consequently, a loss of miR-29 resulted in increased Aβ production [72]. In addition, there are other miRNAs, like miR-195 and miR-107, that target *BACE1* and are deregulated in AD subjects [73,74]. Considering tau protein, miR-132 was previously found to impact tau expression, and its deregulation was discovered in later stage of AD samples [75,76]. Other miRNAs repressing tau synthesis post-transcriptionally include miR-34, miR-107 and miR-219, which were deregulated in brain tissues from AD patients [77,78]. miR125, miR-146a and miR-155 are involved in neuroinflammation and can be also regarded as candidate AD biomarkers [78].

lncRNA (long non-coding RNA) also participated in AD pathology. Revealed by microarray analysis, a total of 315 lncRNAs were significantly dysregulated in the hippocampal tissue of a rat model of AD [79]. BACE1-AS (BACE1 antisense RNA) is an antisense RNA transcribed by RNA polymerase II from the complementary strand of *BACE1*. In terms of activity, BACE1-AS promoted the expression of BACE1 and was directly implicated in the Aβ accumulation [80]. BACE1-AS levels were aberrantly elevated in AD subjects, as well as in APP transgenic mice [80,81]. BACE1-AS was further considered as a potential therapeutic target, as its knockdown reduced amyloid production and plaque deposition in Tg-19959 mice [82]. Two additional lncRNAs involved in Aβ peptide production or accumulation were 51A and 17A, which targeted neuronal sortilin-related receptor gene (SORL1) and GABA B (Gamma-Amino-Butyric Acid B) signaling, respectively [83,84]. Furthermore, lncRNAs associated with synaptic plasticity or apoptosis, exemplified by BC200 (Brain cytoplasmic 200 long-noncoding RNA) and NAT-Rad18, were also suggested as AD biomarkers [85,86].

Circular RNAs (circRNAs) represent a newly evolving group of stable noncoding RNAs abundant in the eukaryotic transcriptome [87]. circRNAs were significantly enriched in human brains, and their relevant network in AD mouse models was involved in Aβ clearance and myelin function [88]. One highly represented circRNA was ciRS-7 (CDR1as), which acted as an endogenous inhibitor of miR-7. miR-7 was highly abundant in human brains and inhibited the activity of ubiquitin protein ligase A (UBE2A), which was involved in clearing toxic amyloid peptides from AD-inflicted brains. Therefore, ciRS-7 might play a causative role in AD pathogenesis though the miR-7-UBE2A pathway. In the hippocampal CA1 region of AD patients, ciRS-7 was dysregulated as confirmed by Northern blotting and sensitive probe RNase R. This alteration may lead to an increased intracellular presence of miR-7, contributing to the occurrence of prototypic cognitive deficits [87]. An alternative mechanism was proposed by Lukiw et al., which stated that ciRS-7 were decreased in brains with AD and acted by reducing protein levels of APP and BACE1 [89]. Furthermore, a recent RNA-sequencing study was performed from individuals with and without AD from the Knight Alzheimer Disease Research Center [90]. The authors quantified an atlas of circRNAs in parietal cortex, establishing significant associations between circRNA and AD diagnosis. Among them, circHOMER1 contains five predicted binding sites for miR-651, which is predicted to target *PSEN1* and *PSEN2* [91]; circCORO1C, which co-expressed with the *APP* and *SCNA* (an AD-related gene), contains two predicted binding sites for miR-105, an miRNA also shown to target *APP* and *SNCA* (α-synuclein). While these bioinformatic results implicated some circRNAs in AD pathogenesis, they still require functional validation in future investigations [90].

### 2.4. Lead Exposure and Epigenetics

Lead is known as an epigenetic modifier [92], with its association to neural epigenetic reprogramming long established. Chronic lead exposure results in various epigenetic changes, characterized by DNA methylation [93], histone modification [94] and ncRNAs [95]. In this subsection, our aim is to summarize lead-induced alterations of the epigenetic molecules which were previously associated with AD pathogenesis (Table 1).

Considering global CpG methylation changes, Senut et al. revealed that lead disrupted global DNA methylation in embryonic stem cells and alters their neuronal differentiation [93]. For an epidemiological survey, it was shown by Pilsner et al. that maternal bone lead levels were associated with DNA methylome in the umbilical cord blood leukocytes of the offspring [117]. Additionally, lead exposure can alter the expression of proteins involved in DNA methylation, including DNA methyltransferase and methyl-cytosine-phosphate-guanine (Me-CpG) binding protein-2 (MeCP2) [116,118].

Except from global profiles, lead also altered methylation level at AD-related genetic loci. By examining 5-mC profiles of DNA extracted from dried blood spots in a cohort of 43 children, Sen et al. identified a series of female-specific methylation changes on stress response genes like *APP* [119,120]. In differentiated SH-SY5Y cells exposed to lead, protein expression was examined six days after the exposure ceased. The findings unveiled that DNMT1 and DNMT3a were significantly downregulated, accompanied by a latent elevation of AD biomarkers SP1 and SP2 (specific protein 2) [22]. No direct proof linked lead exposure and methylated changes at *BACE1*; however, SERBP2 (sterol regulatory element binding protein 2)-BACE1 pathway was shown to be activated in response to developmental lead exposure in rat brains [96]. Moreover, *BACE1* promoter was CpG rich and its expression was closely related to methylation changes [121]. For 5-hmC, Sen et al. profiled lead-associated 5-hmC changes in a human embryonic stem cell model, as well as in umbilical cord blood DNA from 48 mother–infant pairs in a cohort study. According to the results, lead affected 5-hmC level in a set of clusters of cytosine sites, and these sites can be classified into sex-dependent and -independent categories [98].

In terms of histone modification, Luo et al. published an article in 2014 describing an increased acetylated form of histone H3 in rats exposed by 5 or 25 mg/L of lead. This alteration was in parallel with the enhanced transcription of p300, a typical histone acetyltransferase [99]. In another case, perinatal lead exposure reduced H3K9ac level in aging mice, suggesting a lasting effect of H3K9ac on murine physiology [5]. In murine hippocampi exposed by lead, H3K9/14ac was gradually reduced as exposure prolonged, factored by sex and prenatal stress [102]. An additional investigation found that early-life lead exposure induced latent increased expression of H4K12ac and H4K8ac, showing an epigenetic target shared with AD [9]. In the case of HDAC2, our lab previously identified an aberrant increase upon lead exposure in vivo and in vitro. Mediated by knockdown plasmid and chemical inhibitors, HDAC2 blockage led to significant restoration of H3K9ac quantity as well as spatial memory loss [100].

Compared to acetylation, histone methylation is relatively stable and more likely to be linked with long-term lead neurotoxicity. In an animal study, H3K9me3 displayed a stable alteration in rats treated with lead, and cases varied depending on the studied brain regions and genders [5]. Considering H3K27me3 and H3K4me3, in cultured hippocampal neurons, 5 μM of lead exposure resulted in a decreased global level of H3K27me3 at 14 DIV (days in vitro), while no significant changes were observed regarding protein level of H3K4me3. Still, gene promoters enriched with bivalent marks were dysregulated in response to lead invasion, like *Wnt9b* and *Wnt6*, in which H3K4me3 and H3K27me3 displayed divergent alterations [101]. Along with AD-related occasions, this observation may implicate bivalent genes in the pathogenesis of lead-induced AD. Besides, no direct relations were established between lead neurotoxicity and H3S10p (phosphorylation of H3S10) or other types of histone modifications. Interestingly, SUMOylation of EZH2 (a methyltransferase leading to H3K27me3), was deregulated in PC-12 cells due to lead exposure, according to our unpublished data.

In terms of microRNAs revealed by a study, at least seven miRNAs were altered pertaining to their expression levels in mice chronically exposed to 0.2% lead acetate, including upregulated miR-204, miR-211, miR-448, miR-449a, miR-34b and miR-34c, as well as downregulated miR-494 [102]. Considering miRNAs previously suggested to relate Alzheimer’s Disease, Masoud et al. discovered, from various growth stages, that the expression of miR-106b (binding *APP* mRNA) was transiently stimulated, and a similar tendency was observed regarding miR-29, a molecule targeting *SP1*. In addition, miR-29b and miR-132, which were deregulated in AD samples, displayed a profound elevation in a short exposure period [102]. At PND (postnatal day) 700, the level of miR-124 (binds to *SP1* mRNA) was significantly reduced, indicating a potential link with AD symptoms. In another instance, Bollati et al. evaluated miRNA expression when 63 workers at an electric furnace steel plant were incorporated [103]. According to data from blood leukocytes collected following three days of work, miR-222 expression was positively correlated with lead exposure (β = 0.41, *p* = 0.02); miR-146a did not show a significant difference from the control group (control = 0.61 ± 2.42, exposure = 1.90 ± 3.94, *p* = 0.19), but was negatively correlated with blood lead levels (β = –0.51, *p* = 0.011).

Very few studies were performed to investigate insight with relations of lncRNA and lead neurotoxicity. One striking example is lncRNAL20992, which was identified as a key response towards lead neurotoxicity [104]. The authors found that lncRNAL20992 was aberrantly upregulated in a lead-induced neuronal injury model; however, no information was given concerning importance of this lncRNA in AD pathogenesis. Another example is Uc.173, which was significantly decreased in a lead-exposed population and animal model, as well as in in vitro cell lines [122]. Considering roles of AD-related lncRNAs, such as BACE1-AS, 51A, 71A and BC-200, no direct links with lead treatment have been reported to date. Besides, it also remains to be understood whether circRNAs, particularly those dysregulated in AD etiology, can have relevance with chronic lead neurotoxicity.

### 2.5. Epigenetic Mechanisms Linking Lead with AD

By integrating epigenetic data associated with lead or AD, it could be summarized that some key epigenetic determinants were potentially implicated in lead-induced development of AD (Table 1). However, there are a paucity of direct evidence involving epigenetic mechanisms in the studied pathogenesis. In light of this, Zawia group contributed substantially to the relevant research in primate models [9,10,31,102]. Among them, Wu et al. carried out a long-term exposure study based on a cohort of female monkeys randomly grouped and exposed with 1.5 mg/kg/d of lead acetate from birth until 400 d of age. The monkeys were terminated 23 years later, and brain tissues were collected for further examination [31]. Early lead exposure resulted in typical AD-like pathology in aging animals, characterized as intracellular distribution of Aβ and amyloid plaques in the frontal association cortex. Meanwhile, methylation activity of DNMT1 in the brain tissues derived from developmental rats was reduced by 20%, a phenomenon confirmed in mouse primary neurons [31]. The inhibition of DNMT1 may account for a concomitant lower methylation at the promoter of *APP* [123]. Meanwhile, authors examined the abundance of other epigenetic markers in the cerebral cortex of 23-year-old primates, showing that developmental lead exposure led to a decreased expression of MeCP2 (*p* < 0.05), DNMT1 (*p* < 0.001) and DNMT3a (*p* < 0.001), along with marked increases in the expression of H3K4me2 (*p* < 0.01) as well as H3K9ac, H4K8ac and H4K12ac (*p* < 0.001) [9]. An analogous study was performed using C57BL/6 mice as research subjects, where male pups received 0.2% lead acetate from PND 1 through PND 20, and subsequently brain samples were collected across the lifespan till PND 700. Chromatin immunoprecipitation sequencing revealed that exposure to lead resulted in a global downregulation of H3K9ac, however, in the absence of enrichment on genes associated with the Alzheimer’s pathway. This might indicate that AD genes were not readily to be controlled by H3K9ac in the studied context [106].

Negative correlation between methylation and gene expression is supposed to consolidate the epigenetic hypothesis in lead-induced AD. To this end, Alashwal et al. combined gene expression and methylation data in multiple AD studies with lead-exposed origin [124]. Data integration revealed a significant inverse correlation between gene expression and site-specific methylation, which substantiates roles of epigenetic regulators. Besides, due to its inhibitory activity against sequence-complementary mRNAs, target genes of unique microRNA can be readily predicted, rendering their roles in lead-induced AD relatively reliable, albeit without experimental validation in some cases. For example, Dash et al. observed an increase in miR-34c in the context of lead exposure, and this microRNA regulates tau gene expression [125]. Furthermore, miRNAs impacted by lead included those targeting DNMTs, MeCP2 or proteins involved in histone modification [102,126], suggesting an interlaced network of epigenetic regulators participating in lead-induced AD pathogenesis.

## 3. Parkinson’s Disease

### 3.1. PD and Risk Genes

Parkinson’s disease (PD) is the second most common neurodegenerative disorder, affecting about 2–3% of population worldwide [127]. PD symptoms were mainly characterized by a loss of dopaminergic neurons in the substantia nigra pars compacta (SN), presence of Lewy bodies, as well as classic motor dysfunction [128,129]. PD might originate from genetic or environmental cues, whereas more than 90% cases are sporadic and arise from gene–environment interactions, enabling a possible involvement of epigenetic mechanisms [130,131].

Regarding risk genes associated with PD, genetic mutations leading to inherited form of PD should be underpinned. Conventionally, PD can arise from dominant mutations in α-synuclein (*SNCA*), leucine-rich repeat kinase 2 (*LRRK2*), and vacuolar protein sorting-associated protein 35 (*VPS35*) genes, as well as recessive mutations in parkin (*PARK2*), phosphatase and tensin homolog-induced putative kinase 1 (*PINK1*), and DJ-1 (*PARK7*) genes [130,131]. Besides, the most common risk factor is mutation in the gene *GBA1*, which encodes for glucocerebrosidase (GCase) [128]. GCase is responsible for hydrolysis of glucosylceramide to glucose and ceramide. Approximately 7–10% of PD patients harbor a mutation in *GBA1* [132].

### 3.2. Lead and PD Etiology

Increased PD risks have been associated with a number of environmental factors, ranging from rotenone, paraquat, pesticides and traumatic brain injury to toxic metals represented by lead [133,134,135]. In an epidemiological survey, the authors discovered that, compared to the lowest quartile of bone lead levels, the odds ratio for PD in the highest quartile was 3.21 (95% CI, 1.17–8.83), indicating that cumulative lead exposure increased the risk of PD [136]. Prior to this finding, in 2006, Coon enrolled 121 PD patients and 414 age-, sex-, and race-, frequency-matched controls in a case-control study. Based on occupational data on participants from 18 years of age, whole-body lifetime exposures were established and used to estimate risk factors of PD. According to the results, the risk of PD was elevated by >2-fold for individuals in the highest quartile for lifetime lead levels relative to the lowest quartile [137]. These studies gave clear population-based evidence to support the relevance of long-term lead exposure with PD occurrence. Compared to AD, in vitro valid PD models were scarcely appreciated based on lead treatment, while conventionally, MPTP (1-methyl-4-phenyl-1,2,3,6-tetrahydropyridine), a neurotoxin inhibiting mitochondrial complex I, was used to form in vivo and in vitro PD models [138].

### 3.3. Epigenetics and Lead-Induced PD

Due to the lack of efficient PD models based on lead exposure, epigenetic implication in lead-induced PD is rarely clarified. Still, epigenetic regulators played roles in PD etiology, some of which may constitute common mechanisms shared by lead neurotoxicity. In a blood and brain measurement of PD patients, differential methylation patterns were observed in a range of genes compared to age-matched controls, and many of these genes were previously associated with PD [107]. Interestingly, one of the major genes affected is *HLA-DQA1* (a HLA allele located in the extended major histocompatibility complex on chromosome 6), which was also regulated by HDAC1 overexpression [107]. In a *Drosophila* model of PD, it demonstrated that α-synuclein bound directly to histones, inhibited acetyltransferase activity and reduced H3Ac levels [108]. Cases were different in a human primary motor cortex study, which revealed a decrease of H3K9ac, as well as increase of H3K14ac and H3K18ac, leading to a net hyperacetylation of histone H3 [109]. It is noteworthy that a reduced H3K9ac level was a consensus epigenetic event shared by lead neurotoxicity in rats [10,100]. Another example showed that *SNCA* promoter was hypomethylated in brains of PD patients relative to normal tissues [110]. This methylation mark was found to influence gene expression in cultured cells and regarded as an effective biomarker for PD. SNP variants of *SNCA* gene potentially decided lead-induced risk of PD, specifically, lead increased PD odds only among subjects carrying non-deleterious *SNCA* allele, while subjects with highly susceptible *SNCA* alleles were less affected [111]. This epigenetic information was summarized in Table 1.

## 4. Amyotrophic Lateral Sclerosis

Amyotrophic lateral sclerosis (ALS) is another prevalent neurodegenerative disease characterized by the selective loss of upper and lower motor neurons [112]. ALS is classified into two subtypes: familial and sporadic, wherein sporadic disease represent 90% of cases. Genes associated to ALS include superoxide dismutase 1 (*SOD1*), chromosome 9 open reading frame 72 (*C9orf72*), fused in Sarcoma (*FUS*), and TAR DNA binding protein 43 (*TDP-43*) [139]. Lead is a risk factor contributing to ALS pathogenesis. Kamel et al. conducted a case-control study in New England, USA, with 109 ALS cases and 256 population-based controls [140]. As shown by the results, ALS was associated with blood and bone lead levels. Bone lead levels were more reliable to characterize disease risks, as they exhibited a 2.3- to 3.6-fold increase in risk for each doubling of bone lead relative to the 1.9-fold increase for each g/dl increment of blood lead level [140]. This epidemiological evidence was consistent with previous reports [141,142], and summarized in a recent meta-analysis [143].

Compromised by the lack of animal models of lead-induced ALS, studies concerning epigenetic roles in this pathogenesis are lacking. However, epigenetic factors were considerably implicated in ALS pathogenesis. Observations in cellular models of ALS unveiled an upregulation of DNMTs and 5-mC. Similar results were derived from human ALS motor neurons [14]. As shown previously, DNMTs were also affected by lead exposure in nervous tissues. For example, in a 23-year-old primate with early exposure of lead, protein levels of DNMTs were significantly affected [9]. Another report found a dysregulated DNA methyltransferase activity in mouse cortical neuronal cells exposed to lead [31]. In terms of histone modification, mice carrying ALS-linked SOD1 mutation displayed reduced Sirt1 (a histone deacetylase) level in the spinal cord, while an opposite tendency was observed in muscle tissue with progression of the disease [144]. Sirt1 was also an epigenetic target of lead in the rat hippocampus, with its phosphorylation inhibited by this neurotoxic process [145]. Besides, a range of microRNAs were involved in ALS pathogenesis, including miR-155, miR-142, miR-409, miR-495, miR-388, miR-206, has-miR4299, etc. [112]. Basically, these miRNAs targeted genes with relevance to neuronal functioning, as exemplified by ubiquilin 2 (*UBQLN2*), the RNA binding protein Fox-1 (*RBFOX1*), reelin (*RELN*), *Gria2* (encoding glutamate ionotropic receptor AMPA type subunit 2), as well as *EPHA4* (ephrin type-A receptor 4, responsible for brain development and neuronal migration) [112]. Among these miRNAs, miR-142 exhibited a positive correlation with increasing tibia lead levels, although this trial was not performed in nervous tissues [113]. The relevant information was shown in Table 1.

## 5. Attention Deficit/Hyperactivity Disorder

Apart from neurodegenerative disorders, chronic lead exposure can also induce other forms of neurological disease, like Attention deficit/hyperactivity disorder (ADHD). ADHD is one of the most commonly diagnosed psychiatric illnesses in children [146]. The global statistics show that more than 10% of the population has ADHD, making this neurodevelopmental disease an important health issue [147]. Lead exposure is regarded as one of major factors contributing to ADHD, as numerous studies connected them in populations with varying regions and ethnicities [148]. According to these surveys, even the blood lead level of less than 10 µg/dL may affect children with ADHD or at least one subtype of symptoms.

Concerning epigenetic roles, in a case-control study towards Chinese children, we established an association of ADHD with a specific CpG site methylation at the promoter of *DRD4* gene, as well as with expression levels of histone acetylation-related genes: *HDAC1*, *Myst4* and *p300* [114]. Moreover, among the same population, blood lead levels in ADHD children were significantly higher than age-/gender-matched controls, therefore, these epigenetic factors may contribute to lead-related pathogenesis of ADHD. The importance of DNA methylation at *DRD4* (dopamine receptor D4) was also demonstrated in a twin study and a longitudinal cohort investigation [149,150]. In another case, MeCP2 expression was found to be reduced in ADHD, revealed by a measurement in the frontal cortex samples [115]. Abnormal MeCP2 expression was also described in an animal study, which showed that lead exposure altered DNMT (DNA methyltransferase) and MeCP2 levels in the hippocampus of exposed dams [116]. Besides, Kandemir et al. evaluated miRNA levels in 52 ADHD patients versus the control group, and a marked dysregulation of circulating miRNA levels were discovered in the ADHD group [151].

## 6. Therapeutics for Neurological Disorders Based on Targeting Epigenetic Molecules

After identifying key epigenetic regulators involved in lead-induced neurological disorders, some therapeutic approaches were then developed to interfere with disease progression. These therapeutics can be classified into two major categories, DNA methylation inhibitors and histone deacetylase inhibitors [152].

The most commonly used inhibitors towards DNA methylation is 5-azacytidine (5-aza-C). 5-aza-C was developed over 40 years ago, which showed strong inhibitory activity against broad range of DNA methyltransferases as a cytosine analog [153]. 5-aza-C was potent in treating various types of cancer cells, through disrupting their cell cycle or sensitizing tumor cells to T cell-mediated cytotoxicity [154,155]. In CNS-related disease, 5-aza-C was primarily used to study roles of DNA methylation in different cellular physiology. For example, the use of 5-aza-C to treat neural stem cells could inhibit DNA methylation, thereby disrupting neuronal migration and differentiation [156]. Meanwhile, extreme caution should be exercised when using this chemical to treat AD patients, due to 5-aza-C, as an antimetabolite, showing an increased frequency of undercondensation of specific chromosomes in AD patients relative to healthy controls [157]. Nevertheless, this approach has been used in some neurodegenerative diseases, including Friedreich’s ataxia and fragile X syndrome [158,159].

A more prevalent epigenetic therapy is the use of HDAC inhibitor (HDACi). Many studies reported that transgenic AD mice treated with HDACi, such as trichostatin A (TSA), valproic acid (VPA), sodium butyrate (NaB) or vorinostat (SAHA), showed an improvement of learning and memory, as well as a reduced presence of Aβ level in transgenic mice [160,161]. TSA was found to induce BDNF expression in Neuro-2a cells. By suppressing HDAC2 expression [162], TSA resulted in a striking recovery of impaired cognitive function [163]. Of note, most HDACis were broad-ranging inhibitors against diverse isoforms of HDAC, and not specifically targeted HDAC2, which was, however, viewed as a key epigenetic molecule involved in the regulation of learning and memory [164]. In our previous investigation, lead can cause a significant elevation of HDAC2 level in either developmental rats or neuronal cell models, along with an impairment of spatial memory. When HDAC2 was suppressed by TSA or shHDAC2, a specific siRNA construct, lead neurotoxicity was counteracted, and the damaged spatial memory was improved. This is a direct proof that HDAC2 is implicated in lead-induced memory deficits, meanwhile demonstrating the efficacy of HDAC inhibitors [100].

For other therapeutics, VPA was found to decrease Aβ production in transgenic mice, improving their behavioral performance [165]. In another instance, VPA could attenuate latent Aβ accumulation and HDAC abnormality induced by lead in the SH-SY5Y cell line [166]. In terms of SAHA, in the APPswe/PS1dE9 AD mouse model, SAHA showed robustness in restoring contextual memory by inhibiting HDAC6 at an early stage of AD [60]. Relative to SAHA, it seems that NaB tended to play a crucial part at a later stage of AD; Govindarajan et al. reported that NaB alleviated the associative memory deficit in APPPS1-21 mice when administered at a very advanced stage of pathology [167]. Interestingly, butyrate is an important metabolite of intestinal microbiota, and alters depending on core microbial composition [168]. Therefore, this HADC inhibitor can potentially mediate a beneficial effect of some food ingredients on psychiatric disorders.

In addition, to counteract the negative effect of lead, chelation therapy is found to reduce the body burden of lead. However, this intervention strategy is only recommended for children with BLL > 45 µg/dL [1]. The chelation strategy was proven ineffective in treating low-level exposures to lead, and failed to reverse the cognitive and behavioral deficits caused by lead exposure with long-term/chronic pattern [169,170]. Therefore, it is proposed that treatment strategies directed to the neuronal representations may be more effective in alleviating the lead-induced neurological damages [1]. In one of our previous publications [171], probiotic formulation effectively mitigated lead-led memory deficits by remodeling hippocampal histone modifications in SD rats, which demonstrated the feasibility of epigenetic manipulations in treating lead-led neuronal impairment.

In summary, histone deacetylase was crucial epigenetic determinant connecting early lead exposure to progressive course of various neurological disorders, thereby becoming an optimal candidate for pharmaceutical intervention.

## 7. Conclusions and Future Perspective

In conclusion, epigenetic factors play essential roles in mediating lead-induced neurological disorders. In particular, some epigenetic marks are rather stable or can even persist into the next generation, giving rise to long-term inhibition of gene expression. This event accommodates “fetal programming, late onset” manifestation of multiple neurodegenerative diseases, and highlights the prime importance of epigenetic regulators in pathogenesis and intervention of CNS-related diseases, in which various forms of HDAC inhibitors are largely implicated. As a newly emerging research field, epigenetics are anticipated to have several challenges and future directions regarding their roles in lead-induced neurological diseases: (I) a key challenge is to understand if epigenetic changes are a cause or an effect of the pathological process. Currently, most relevant studies have focused on examining epigenetic alterations during disease progression, and these co-existing events did not clarify cause–consequence relations. Establishing the cause–effect relations of epigenetic changes and pathological process is a prerequisite to judge the potential applicability of epigenetic intervention therapy. Intervention studies using inhibitors or RNAi (RNA interference) constructs were scarcely applied in the long-lasting period spanning the development of AD or other neurodegenerative diseases; (II) except AD, very few animal models were established by lead exposure to accurately characterize key symptoms of PD, ALS or ADHD. This situation circumvented efforts to define epigenetic roles in these neurotoxic processes, because an appropriate disease model is essential in translating data derived from animal studies into human bodies. In other aspects, given the difficulty of obtaining human-based samples, an animal model accurately describing diseases may facilitate the molecular studies involved in the lead-induced neurological disorders; (III) new epigenetic mechanisms, such as circRNA, lncRNA and 5-hmC, need to be underscored regarding their associations with neurological disorders of environmental origin. These missing epigenetic mechanisms should only be clarified prior to development of any intervention strategies; (IV) the current epigenetic therapeutic tools, like HADC inhibitors or CpG methylation inhibitors, can only be used to treat the adverse neural consequences with these specific epigenetic aberrations. In order to reverse changes of other epigenetic regulators, the corresponding antagonists or agonists are warranted to be developed to interfere with disease progression, based on broader range of epigenetic regulators, like histone methylation and ncRNAs. Besides, other factors, like diet or stress, may predispose some individuals more than others to epigenetic changes. This complicated the direct link of lead with disease, as well as the *“bona fide”* epigenetic mechanisms involved.

## Figures and Tables

**Table 1:** Summary of studies of epigenetics involved in lead-induced neurological disorders.

Disease	Epigenetics	Observations	Associations with Lead
AD	Global DNA methylation	Reduced DNA methylation in human postmortem studies (*n* = 20) [33]	Global DNA methylation reprogrammed in ESCs (*n* = 7) [93]
AD	CpG methylation at *PSEN1*, *BACE1*	Deregulation of methylation status in cultured cells (*n* = 4) [39]	SERBP2-BACE1 pathway activated in rat brains (*n* = 40) [96]
AD	CpG methylation at *TREM2*, *BDNF*	Differentially methylated in multiple models (*n* = 20; *n* = 30; *n* = 506, respectively) [46,47,48]	Positive relations between BDNF expression and umbilical cord blood lead level (*n* = 60) [97]
AD	Global 5-hmC level	Elevated 5-hmC levels in preclinical AD subjects (*n* = 30) [53]	5-hmC levels at a set of cluster CpG sites affected in umbilical cord blood DNA (*n* = 48) [98]
AD	H4K12ac (acetylation of histone H4 at lysine 12)	Early increase in transgenic mouse model (*n* = 19) [56]	Latent increase of H4K12ac expression in aging primate brains (*n* = 5) [9]
AD	H4 and H3 acetylation	Hypoacetylation of H4, not H3, in tg2576 mice [58]	H3 acetylation increased in developmentally exposed rats (*n* = 3) [99]
AD	HDAC2 (histone deacetylase 2)	Elevated HDAC2 levels in AD patients (*n* = 6–9) [59]	HDAC2 aberrantly increased in developmentally exposed rats (*n* = 3) [100]
AD	H3K9me (methylation of histone H3 at lysine 9)	Increase with age in the 3xTg-AD mouse model and AD model neurons (*n* = 6) [63]	Stable alteration depending on brain regions and genders in rats (*n* = 7–10) [5]
AD	H3K4me3/H3K27me3 (trimethylation of histone H3 at lysine 4)/ trimethylation of histone H3 at lysine 27)	Identified DMRs overlapped promoters with bivalent markers in genome-wide methylation study in AD brains (*n* = 34) [33]	Bivalent regulation of *Wnt9b* and *Wnt6* altered in hippocampal neuronal culture (*n* = 3) [101]
AD	H3S10p (phosphorylation of H3S10)	Hyperphosphorylation in AD hippocampal neurons (*n* = 17) [66]	No direct link with lead was identified.
AD	SUMOylation (SUMO, small ubiquitin-like modifiers)	SUMOylation of HDAC1 was a protective mechanism against Aβ toxicity in mouse model (*n* = 5) [67]	SUMOylation of EZH2 deregulated in lead-exposed PC-12 cells (unpublished data)
AD	miR-29	Reductions in AD patients, along with a increment of its target, BACE1 protein level (*n* = 5) [72]	MiR-29 elevated in short exposure period in developmentally exposed mice (*n* = 3) [102]
AD	miR-132	Deregulation of miR-132, targeting tau expression, in later stage of AD samples (*n* = 90; *n* = 7, respectively) [71,72]	miR-132 increased in short exposure period in developmentally exposed mice (*n* = 3) [102]
AD	miR-146a	Deregulation of miR-146a, involved in neuroinflammation, in brain tissues from AD samples (*n* = 6) [77,78]	miR-146a negatively correlated with blood lead levels in 63 workers, but not significant (*n* = 63) [103]
AD	BACE1-AS (BACE1 antisense RNA)	Aberrant elevation of BACE1-AS, promoting expression of BACE1, in AD subjects and APP transgenic mice [76,77]	No correlation of BACE1-AS with lead neurotoxicity was reported to date.
AD	lncRNAL20992	No information was given concerning importance of lncRNAL20992 in AD pathogenesis	lncRNAL20992 was aberrantly upregulated in a lead-induced neuronal injury model (*n* = 3) [104]
AD	ciRS-7 (CDR1as)	Dysregulation in hippocampal CA1 region of AD patient, contributing to increased level of miR-7 and AD pathogenesis [87]	No associations were established.
AD	circHOMER1	Significant associations with AD diagnosis, as measured in an RNA-sequencing study. circHOMER1 contained five predicted binding sites for miR-651, which is predicted to target *PSEN1* and *PSEN2* (*n* = 77) [105]	No associations were established.
AD	circCORO1C	Significant associations with AD diagnosis, as measured in an RNA-sequencing study. circCORO1C contained two predicted binding sites for miR-105, which is predicted to target *APP* and *SCNA* (*n* = 77) [105]	No associations were established.
AD	MeCP2 (Methyl-CpG Binding Protein 2), DNMT1, DNMT3a (DNMT, DNA (cytosine-5)-methyltransferase), H3K4me2, H3K9ac, H4K8ac, H4K12ac	In a cohort of female monkeys randomly grouped and exposed with 1.5 mg/kg/d of lead acetate from birth till 400 d of age, cerebral cortex was sampled from 23-year-old primates, which exhibited AD symptoms. Developmental lead exposure led to a decreased expression of MeCP2 (*p* < 0.05), DNMT1 (*p* < 0.001) and DNMT3a (*p* < 0.001), along with marked increase in the expression of H3K4me2 (*p* < 0.01), as well as H3K9ac, H4K8ac and H4K12ac (*p* < 0.001) (*n* = 5; *n* = 4, respectively) [9,31]	
AD	H3K9ac	Male C57BL/6 mice received 0.2% lead acetate from PND 1 through PND 20, and subsequently brain samples were collected across the lifespan till PND 700. Global downregulation of H3K9ac was observed, and chromatin immunoprecipitation sequencing revealed distinct subsets of H3K9ac-enriched genes (*n* = 5) [106]	
PD	DNA methylation at *HLA-DQA1* (Major Histocompatibility Complex, Class II, DQ Alpha 1)	Deregulation of methylation levels at *HLA-DQA1* in blood and brain measurement of PD patients. HLA-DQA1 was also regulated by *HDAC1* (*n* = 5) [107]	Developmental lead exposure changes the function of HDAC1/2 complex in rats and PC-12 cells (*n* = 3) [100]
PD	H3 acetylation	Reduced acetyltransferase activity and H3Ac level in a *Drosophila* model of PD [108]; net hyperacetylation of histone H3 in human primary motor cortex (*n* = 3) [109]	H3 acetylation increased in developmentally exposed rats (*n* = 3) [99]
PD	H3K9ac	Decreased H3K9ac level in human primary motor cortex (*n* = 9) [109]	H3K9ac decreased in lead-exposed PC-12 cell and rat hippocampus (*n* = 3) [100]
PD	DNA methylation at *SCNA* promoter	Hypomethylation at *SCNA* promotor in brains of PD patients (*n* = 12) [110]	Lead increased PD odds only among subjects carrying non-deleterious *SCNA* allele (*n* = 328) [111]
ALS	DNMT/5-mC	Upregulation of DNMTs and 5-mC in cellular models of ALS, as well as human ALS motor neurons (*n* = 5) [9]	Protein levels of DNMTs significantly affected in a 23-year-old primate with early life exposure of lead (*n* = 5) [9]
ALS	miR-142	Upregulation of miR-142 levels in the spinal cords of ALS patients, possibly by targeting cell death or brain development-related pathway [112]	miR-142 exhibited a positive correlation with increasing tibia lead levels in the cervix tissue (*n* = 45) [113]
ADHD	CpG 1 methylation at *DRD4* (dopamine receptor 4) promoter, HDAC1, Myst4 (histone acetyltransferase 4), p300	In a case-control study towards Chinese children, ADHD was associated with a specific CpG site methylation at the promoter of *DRD4* gene, as well as with expression levels of histone acetylation-related genes: *HDAC1*, *Myst4* and *p300*. Among the same population, blood lead levels in ADHD children were significantly higher than age/gender-matched controls (*n* = 50) [114]	
ADHD	MeCP2	Reduced MeCP2 expression in ADHD frontal cortex samples (*n* = 5) [115]	Lead exposure altered DNMT and MeCP2 levels in the hippocampus of exposed dams (*n* = 6–8) [116]

AD, Alzheimer’s disease; PD, Parkinson’s disease; ALS, Amyotrophic lateral sclerosis; ADHD, Attention deficit/hyperactivity disorder.
